# Analysis of 2,440 human exomes highlights the evolution and functional impact of rare coding variation

**DOI:** 10.1186/gb-2011-12-s1-i1

**Published:** 2011-09-19

**Authors:** Joshua Akey

**Affiliations:** 1 Department of Genome Sciences, University of Washington, Seattle, WA 98195, USA

## 

Deep exome resequencing is a powerful approach for delineating patterns of protein-coding variation among genes, pathways, individuals and populations. We analyzed exome data from 2,440 individuals of European and African ancestry as part of the National Heart, Lung, and Blood Institute’s Exome Project, the aim of which is to discover novel genes and mechanisms that contribute to heart, lung and blood disorders. Each exome was sequenced to a mean coverage of 116×, allowing detailed inferences about the population genomic patterns of both common variation and rare coding variation. We identified more than 500,000 single nucleotide variations, the majority of which were novel and rare (76% of variants had a minor allele frequency of less than 0.1%), reflecting the recent dramatic increase in the size of the human population. The unprecedented magnitude of this dataset allowed us to rigorously characterize the large variation in nucleotide diversity among genes (ranging from 0 to 1.32%), as well as the role of positive and purifying selection in shaping patterns of protein-coding variation and the differential signatures of population structure from rare and common variation. This dataset provides a framework for personal genomics and is an important resource that will allow inferences of broad importance to human evolution and health.

